# Bridging Gaps in Cancer Pain Care: Barriers, Solutions, and a Path Forward for Integrated Management

**DOI:** 10.3390/curroncol32110610

**Published:** 2025-11-01

**Authors:** Marta Gentili, Francesco Cellini, Leonardo Consoletti, Massimo Di Maio, Diego M. M. Fornasari, Gianpaolo Fortini, Marco Krengli, Ernesto Maranzano, Silvia Natoli, Stefano Pergolizzi, Rodolfo Sacco, Luca Giacomelli

**Affiliations:** 1 Fondazione Nora e Alberto Gentili ETS, 20010 Milan, Italy; mmc.gentili@gmail.com; 2 Dipartimento di Diagnostica per Immagini, Radioterapia Oncologica ed Ematologia, Fondazione Policlinico Universitario “A. Gemelli” IRCCS, 00168 Roma, Italy; f.cellinimd@gmail.com; 3 Dipartimento Universitario Diagnostica per Immagini, Radioterapia Oncologica ed Ematologia, Università Cattolica del Sacro Cuore, 20123 Roma, Italy; 4 Pain Medicine Unit, Department of Medical and Surgical Sciences, University Hospital of Foggia, 71121 Foggia, Italy; leonardoconsoletti@libero.it; 5 Federdolore Società Italiana dei Clinici del Dolore (Federdolore-SICD), 00144 Roma, Italy; 6 Department of Oncology, University of Turin, AOU Città della Salute e della Scienza di Torino, 10126 Turin, Italy; massimo.dimaio@unito.it; 7 Associazione Italiana di Oncologia Medica (AIOM), 20133 Milan, Italy; 8 Department of Medical Biotechnology and Translational Medicine, Università degli Studi di Milano, 20122 Milan, Italy; diego.fornasari@unimi.it; 9 Associazione Italiana per lo Studio del Dolore (AISD), 00193 Rome, Italy; 10 Direttore SC Cure Palliative Integrate ASST Sette Laghi, 21100 Varese, Italy; gianpaolo.fortini@asst-settelaghi.it; 11 Referente di DG Welfare Lombardia per la Terapia del Dolore e Cure Palliative, 20124 Milano, Italy; 12 Società Italiana di Cure Palliative (SICP), 20124 Milan, Italy; 13 Department of Surgical, Oncological and Gastroenterological Sciences (DISCOG), University of Padua, 35129 Padova, Italy; marco.krengli@unipd.it; 14 Division of Radiotherapy, Veneto Institute of Oncology IOV IRCCS, 35128 Padua, Italy; 15 Italian Association of Radiotherapy and Clinical Oncology, 20124 Milan, Italy; 16 Faculty of Medicine and Surgery, University of Perugia, 06129 Perugia, Italy; ernesto.maranzano@libero.it; 17 Department of Clinical-Surgical Diagnostic and Pediatric Sciences, University of Pavia, 27100 Pavia, Italy; silvia.natoli@unipv.it; 18 Unit of Pain Therapy Service, Foundation Istituto di Ricovero e Cura a Carattere Scientifico (IRCCS), Policlinico San Matteo, 27100 Pavia, Italy; 19 Area Culturale Dolore Società Italiana Anestesia, Analgesia, Rianimazione e Terapia Intensiva (ACD SIAARTI), 00184 Rome, Italy; 20 Department of Biomedical, Dental Science and Morphological and Functional Images, University of Messina, 98125 Messina, Italy; stpergolizzi@unime.it; 21 Gastroenterology Unit, Department of Medical and Surgical Sciences, University of Foggia, 73122 Foggia, Italy; saccorodolfo@hotmail.com; 22 Polistudium SRL, 20121 Milan, Italy

**Keywords:** cancer pain, clinical pathways, education, interdisciplinary management, policy advocacy, supportive care

## Abstract

**Simple Summary:**

Cancer pain affects most patients with advanced disease, yet remains undertreated despite available treatments and guidelines. In Italy, a national meeting of cancer care experts identified four main barriers preventing effective pain management: healthcare providers lack adequate training in pain treatment, different medical teams work separately rather than together, professional roles are unclear, leading to confusion about responsibilities, and standardized care protocols are difficult to implement in practice. The experts proposed solutions including better education for doctors and nurses, creating integrated cancer centers where different specialists work together, clarifying who does what in pain management, and developing flexible treatment guidelines that can be adapted to different hospitals. Additionally, patient misconceptions about pain medications need to be addressed through education. These recommendations could improve pain care for cancer patients by ensuring that healthcare teams are better trained, work together more effectively, and follow consistent approaches to pain treatment, ultimately reducing suffering and improving quality of life.

**Abstract:**

Cancer-related pain remains one of the most frequent and burdensome symptoms in oncology, significantly impairing patients’ quality of life and functional status. Despite advances in treatment and the availability of evidence-based guidelines, pain continues to be undertreated worldwide. In Italy, legislative efforts such as Law 38/2010 have not fully translated into consistent clinical practice. On 28 March 2025, a national roundtable held in Rome, Italy, brought together experts from medical oncology, radiation oncology, palliative care, anesthesiology, and pain medicine, representing the main Italian scientific societies involved in oncology and supportive care, to examine the current status of cancer pain management and develop a consensus on actionable priorities. Four key gaps were identified: insufficient education and training of healthcare providers in pain management; fragmented care pathways and limited interdisciplinary integration; lack of clarity regarding professional roles; and challenges in implementing shared diagnostic and therapeutic care pathways (*Percorsi Diagnostico Terapeutici Assistenziali*). The roundtable proposed coordinated strategies to address these gaps, including expanding interdisciplinary educational initiatives and integrating pain management into undergraduate and specialty curricula; establishing local oncology orientation centers to provide joint, patient-centered assessments; promoting cross-specialty collaboration through congress sessions, educational activities, and practical workshops; and developing adaptable therapeutic frameworks to ensure standardized yet context-sensitive care delivery. This congress report formalizes a joint framework aimed at embedding pain management within comprehensive cancer care. Its implementation will require sustained advocacy, structured education, and alignment of clinical practice with policy support. By addressing these barriers through pragmatic, evidence-informed actions, the proposed strategies aim to optimize timely, integrated, and effective pain care, ultimately improving outcomes for patients with cancer.

## 1. Introduction

Cancer-related pain remains one of the most frequent and burdensome symptoms experienced by patients with cancer, significantly impairing their quality of life and functional status. Despite advances in cancer treatment and the availability of evidence-based guidelines, the prevalence of pain remains high, affecting nearly half of all patients during active treatment and over 70% of those with advanced disease, with approximately one-third reporting moderate-to-severe pain intensity [1,2].

In Italy, recent surveys indicate persistent deficiencies in pain assessment, undertreatment, and delayed integration of supportive care into oncology practice, despite legislative initiatives such as Law 38/2010 aimed at guaranteeing access to palliative and pain care [3,4]. Contributing factors include insufficient education in pain management within medical curricula, fragmented care pathways lacking true interdisciplinarity, and persistent misconceptions surrounding opioid use [5,6]. As highlighted globally, these issues are compounded by systemic barriers, including inadequate coordination among specialists and uneven resource allocation [7,8].

Educational initiatives targeting healthcare providers and patients alike have shown promise in improving pain knowledge and adherence to treatment, yet their translation into improved clinical outcomes remains inconsistent [9]. Addressing cancer pain thus requires an integrated approach that transcends discipline-specific silos and embeds pain management as a core component of oncology care [10].

In response to these urgent needs, an institutional roundtable organized by the Fondazione Nora and Alberto Gentili (Nora and Alberto Gentili foundation; https://fondazionegentili.it/) was convened in Rome, Italy, on 28 March 2025, during the Radiopain congress (https://radioterapiaitalia.it/event/radiopain-la-gestione-del-dolore-in-radioterapia-per-una-presa-in-carico-multidisciplinare-del-paziente-oncologico-roma-28-29-marzo-2025/, accessed on 3 October 2025). This roundtable brought together representatives of leading Italian scientific societies involved in oncology and supportive care: the Italian Association of Radiation Oncology (AIRO), the Italian Association of Medical Oncology (AIOM), the Italian Society for the Study of Pain (AISD), the Federdolore–SICD (Italian Society of Pain Clinicians), the Italian Society of Palliative Care, and the Pain and Palliative Care Division of the Italian Society of Anesthesia, Analgesia, Resuscitation and Intensive Care (ACD SIAARTI). This meeting provided a platform for interdisciplinary dialogue to examine persistent gaps in pain management and to develop a shared position with actionable proposals aimed at improving education, integration of care, and institutional advocacy.

### Roundtable Discussion and Participants

To inform the roundtable discussions, a preliminary literature search was conducted focusing on publications addressing cancer pain management challenges and solutions in the Italian healthcare context over the past 5 years. Key search terms included ‘cancer pain’, ‘Italy’, ‘pain management’, ‘palliative care’, ‘oncology’, and ‘healthcare barriers’. This targeted search aimed to identify current evidence specific to the Italian scenario to facilitate expert discussions, rather than constitute a comprehensive systematic review, which was beyond the scope of this initiative.

The institutional roundtable was held on 28 March 2025, in Rome, Italy during the Radiopain national course, bringing together 10 expert representatives from the major Italian scientific societies involved in cancer pain management (see Introduction). The 90-min discussion was moderated by two session leaders (M.G. and F.C.), who were also the initiative’s promoters, and followed a predetermined agenda focusing on four key areas: educational gaps, interdisciplinary communication barriers, professional role clarity, and implementation challenges for standardized care pathways. The session was audio-recorded and fully transcribed to ensure accurate documentation of discussions and consensus recommendations. Five follow-up consultations were conducted remotely over the following months to refine the proposed solutions and prepare this collaborative position statement.

## 2. Current Situation and Main Challenges

Despite clear advances in cancer care, pain management remains a persistent global challenge. This gap between evidence-based recommendations and routine clinical practice is driven by multiple barriers spanning education, clinical organization, and health system infrastructure. The Italian context mirrors these international trends: despite progressive legislation (Law 38/2010) establishing pain relief and palliative care as patient rights, implementation has been uneven, and outcomes remain suboptimal. Understanding the factors that perpetuate these deficiencies—both globally and within Italy—is essential to inform targeted interventions. Key issues include insufficient training in pain medicine, fragmented care pathways, limited interdisciplinary integration, and the difficulty of translating guidelines and diagnostic and therapeutic care pathways (*Percorsi Diagnostico Terapeutici Assistenziali* [PDTAs]) into routine practice. These deficits are particularly pronounced in non-referral centers, including general hospitals and community oncology practices, where specialized pain management expertise may be limited or unavailable. The gap affects both physicians and nurses across the care continuum, senior healthcare professionals showing the most significant knowledge deficits, reflecting the limited exposure to pain management principles during their foundational training decades ago. Furthermore, patients’ misconceptions regarding pain management can play a role.

### 2.1. Insufficient Education in Pain Management

Educational gaps among healthcare providers remain a cornerstone barrier to adequate pain control worldwide. Surveys consistently show that pain management is underrepresented in both undergraduate and specialty training, leaving many clinicians without formal preparation to assess pain systematically, tailor therapy to individual needs, or use analgesic drugs, including opioids, confidently and appropriately [11].

In Italy, these deficits are notable. A national survey involving medical oncologists, radiation oncologists, and nurses revealed that 85% identified insufficient training as the principal obstacle to effective care, with underutilization of validated tools, such as the Edmonton Symptom Assessment System (ESAS) and Douleur Neuropathique en 4 Questions (DN4), linked to both knowledge gaps and time pressures [4]. Furthermore, only 23.5% of respondents had completed formal training in pain or palliative medicine, underscoring the disconnect between recognized needs and current educational structures [4]. The consequences are evident: inconsistent pain assessment, delayed initiation of analgesic therapy, and persistent “opiophobia”, reflecting both prescriber hesitancy and misinterpretation of regulatory frameworks [3].

While initiatives in Italy, such as the creation of specialty schools in palliative medicine and dedicated postgraduate programs, represent important progress, their reach remains limited relative to the demand [10]. This educational gap directly contributes to pain undertreatment, as demonstrated by suboptimal opioid titration, inappropriate analgesic sequencing, underutilization of radiation therapy, and missed recognition of complex pain syndromes. Therefore, addressing education remains a foundational step toward systemic improvement.

### 2.2. Fragmented Care and Lack of Interdisciplinarity

Cancer pain management requires input from multiple disciplines, yet care is often siloed. Internationally, patients frequently experience sequential, non-communicating consultations, resulting in delays in symptom relief and avoidable suffering [12]. Evidence supports early integration of palliative care and pain services within oncology multidisciplinary teams, improving both quality of life and, in some studies, even survival outcomes [13]. However, this integration remains suboptimal in many clinical settings.

In Italy, formal interdisciplinary coordination is limited. Nationwide surveys highlight that pain specialists are often consulted late, primarily in advanced stages, and structured multidisciplinary case discussions are rare [3]. Time constraints, high patient volumes, and insufficient staffing in supportive care exacerbate this fragmentation [10]. Similar findings were reported across Europe [14].

Geographic disparities further compound the issue: while large oncology centers increasingly embed supportive care teams, many regional and community hospitals lack consistent access to specialized pain services, reinforcing inequities in care delivery [3].

### 2.3. Limited Mutual Understanding of Professional Roles

Effective interdisciplinary care is undermined by insufficient clarity in the division of roles among healthcare providers. Studies reveal overlapping responsibilities between medical oncologists, anesthesiologists, and palliative care physicians, with poor coordination leading to inconsistent prescribing and delayed referrals for complex pain [13].

Italian data echo these findings. Only one-third of clinicians reported effective intra-team communication regarding pain care, with role ambiguity identified as a source of duplication and confusion [4]. This role ambiguity is prevalent across both oncology departments and specialized pain control units, affecting physicians, nurses, and allied health professionals at all career levels, suggesting that the issue stems from systemic rather than setting-specific factors. This lack of structured collaboration particularly impedes the timely recognition and management of breakthrough cancer pain or neuropathic components, which often require specialist expertise [3]. For patients, this lack of clarity creates further uncertainty about whom to consult, delaying access to appropriate interventions [10].

### 2.4. Challenges in Implementing National Shared Care Pathways

Shared standardized and integrated clinical pathways, such as PDTAs, are designed to harmonize care, ensure adherence to evidence-based guidelines, and reduce variability in treatment decisions. However, translating these frameworks into clinical practice is complex. Internationally, while guideline adherence has improved in some high-resource contexts, implementation remains inconsistent, hindered by limited staffing, organizational inefficiencies, and uneven resource allocation [11].

In Italy, despite the introduction of Law 38/2010 mandating systematic pain assessment and documentation, surveys reveal that only 26% of clinicians routinely assess pain at each encounter, and just 17% monitor pain on a daily basis during hospitalization [10]. The adoption of PDTAs is further complicated by regional heterogeneity: healthcare delivery is regionally administered, resulting in differences in infrastructure, specialist availability, and even opioid prescribing policies [3].

Furthermore, existing PDTAs often lack flexibility to adapt to real-world constraints. Institutions without embedded pain or palliative care teams struggle to fulfill prescribed steps, and smaller centers frequently lack access to necessary expertise or rapid referral mechanisms. This implementation gap risks reducing PDTAs to theoretical documents rather than practical tools for clinical improvement.

Crucially, successful PDTA implementation requires sustained institutional investment in training, workflow integration (e.g., shared electronic records with embedded pain assessment prompts), and leadership support to ensure alignment across disciplines. Without these enabling conditions, PDTA risk being inconsistently applied, perpetuating the very variability they are intended to reduce. Addressing these barriers represents not only a clinical priority but also a structural and policy challenge requiring coordinated action at institutional, national, and regional levels.

### 2.5. Patient-Related Barriers and Misconceptions

Patient attitudes and beliefs about pain and analgesic medications represent an additional, often underrecognized barrier to effective cancer pain management. Patients frequently harbor misconceptions about opioid therapy, including exaggerated fears of addiction, concerns about tolerance development, and beliefs that pain medication should be reserved for end-of-life care [15]. These misconceptions, often reinforced by societal stigma surrounding opioid use, lead to patient reluctance to report pain accurately, poor adherence to prescribed analgesic regimens, and requests for suboptimal dosing. Furthermore, some patients view pain as an inevitable consequence of cancer that must be endured, contributing to delayed help-seeking and acceptance of inadequate symptom control. Addressing these patient-centered barriers requires structured educational interventions and improved clinician-patient communication to dispel myths, provide accurate information about analgesic safety and efficacy, and engage patients as active partners in their pain management decisions.

### 2.6. Implications

These challenges—educational gaps, fragmented care, poor role integration, and incomplete pathway implementation—collectively perpetuate a cycle of pain undertreatment that affects patients globally and is acutely evident in Italy. They highlight that cancer pain is not merely a pharmacologic problem but a systemic one, requiring interventions that span education, organizational reform, and policy alignment. Recognizing and addressing these interconnected barriers is essential to embed pain management as a fundamental component of comprehensive oncology care. This includes the use of analgesic drugs, interventional maneuvers, and radiation treatment, and requires moving beyond aspirational frameworks toward sustained, equitable implementation.

## 3. Joint Proposals and Commitments

Building on the gaps identified, the following proposals outline coordinated strategies to improve cancer pain management through education, organizational integration, and policy advocacy (Table 1). These measures aim to address the structural, professional, and institutional deficiencies that impede effective pain care and to provide realistic pathways for implementation.

### 3.1. Strengthening Interdisciplinary Education and Training

Educational deficits remain a primary driver of undertreatment in cancer pain, with clinicians citing inadequate preparation as a central barrier [3,4]. To address this, the scientific societies jointly advocate for the expansion of structured, interdisciplinary education programs that equip practitioners with both the theoretical and practical skills required for effective pain management.

First, joint educational initiatives—delivered collaboratively by medical oncology, radiation oncology, pain medicine, and palliative care societies—should be prioritized. Integrating dedicated modules into existing platforms of scientific societies would leverage established structures, enabling wide dissemination of content without duplicating resources. By specifically targeting new graduates and early-career specialists, these initiatives would help embed pain management principles early in training, counteracting entrenched gaps highlighted in national surveys [4].

Second, undergraduate and postgraduate medical curricula must formally integrate pain medicine and palliative care. The lack of structured training in these areas directly contributes to incomplete understanding and poor adherence to guidelines, as well as inconsistent prescribing of analgesics and underutilization of radiation therapy [6,11]. National advocacy for curricular reform, aligned with international models that embed pain competencies into core requirements for medical and nursing programs, would institutionalize pain education. This shift would move pain management from optional enrichment to a foundational element of clinical training.

Furthermore, educational programs must incorporate training on emerging therapeutic modalities including neuromodulation techniques (such as spinal cord stimulation and intrathecal delivery systems), novel pharmacological agents, and evidence-based integrative therapies. By ensuring that continuing education platforms regularly update content to reflect therapeutic innovations, clinicians can maintain current knowledge and offer patients access to the full spectrum of available pain management options, preventing the lag time between new treatment introduction and clinical integration.

Finally, interdisciplinary workshops and case-based training should be promoted to bridge knowledge silos between specialties. These formats not only improve pain-specific knowledge but also foster practical collaboration and communication skills critical to effective team-based care [9]. By reinforcing a shared lexicon and mutual understanding, they address the role confusion that undermines coordinated management [13].

### 3.2. Establishment of Local Integrated Oncology Orientation Centers

Fragmentation of care is a recurring barrier to effective pain management, with patients often navigating sequential, uncoordinated consultations that delay symptom relief [12,14]. To overcome this, we propose the development of “oncology orientation centers” serving as hubs for joint assessment and coordinated decision-making.

These centers would assemble multidisciplinary teams, including medical oncologists, radiation oncologists, pain physicians, and palliative care specialists, to provide patients with integrated assessments and unified treatment plans. This model aligns with evidence showing that early integration of supportive care within oncology improves quality of life, treatment adherence, and, in some cases, survival [13]. By consolidating expertise into a single point of access, such centers would reduce delays and duplication while fostering a more patient-centered approach to pain.

In addition, regular multidisciplinary meetings should be institutionalized within these centers. Scheduled joint sessions would enable coordinated review of complex cases, ensuring that analgesic strategies, antineoplastic plans, and supportive care measures are aligned. This approach would address not only the fragmentation observed in Italian practice [3] but also the poor communication among teams, which clinicians themselves report as a significant barrier [4].

Crucially, these centers must be designed to be scalable and adaptable to local resources. In larger general hospitals and cancer centers, full-time embedded teams may be feasible, while in smaller or rural settings, virtual multidisciplinary consultations could extend expertise across regions. Digital health platforms enabling teleconsultation have already demonstrated potential to expand access to specialized care and could be harnessed to support such networks [11].

### 3.3. Promoting Mutual Understanding and Collaboration Among Specialties

Confusion regarding the roles and responsibilities of different specialists in pain management undermines both efficiency and care quality. This lack of mutual understanding contributes to delayed referrals, inconsistent prescribing practices, and duplication of effort [3,13].

To address this, structured cross-disciplinary exchanges must become routine. Joint sessions at national and regional congresses—involving medical oncologists, radiation oncologists, anesthesiologists, palliative care physicians, nurses, and allied professionals—would clarify role boundaries and foster collaborative approaches. Embedding pain-focused panels or tracks within major medical and radiation oncology conferences (e.g., AIOM and AIRO) would normalize pain discussions within disease-centered forums, reinforcing its relevance as a core component of cancer care.

Additionally, practical workshops using real-world case discussions should be employed to model interdisciplinary problem-solving. Case-based formats are particularly effective in breaking down hierarchies and encouraging dialogue between specialties, facilitating better understanding of competencies, referral triggers, and shared decision-making pathways [4]. Over time, these exchanges can reduce the variability that arises when clinicians operate in isolation and support the cultural shift toward collaborative, patient-centered care.

Interdisciplinary professional development also has a multiplier effect. By equipping generalists and oncologists with core pain management competencies while clarifying when specialist involvement is warranted, it ensures efficient resource utilization, preventing bottlenecks and unnecessary delays in referral [11].

### 3.4. Creating Shared, Adaptable Therapeutic Frameworks

Finally, while guidelines such as those from the European Society for Medical Oncology (ESMO) and AIOM provide robust, evidence-based recommendations [11], their translation into uniform practice remains inconsistent. In Italy, regional heterogeneity in resources and policies complicates the elaboration and application of standardized PDTAs, with surveys highlighting wide variation in pain assessment and opioid prescribing [3,10].

To bridge this gap, we propose drafting therapeutic frameworks that combine evidence-based principles with pragmatic flexibility. These frameworks would define the fundamental standards for assessment, pharmacological management, interventional procedures, radiotherapy, and referral processes, while allowing for adaptation to local infrastructure and staffing levels. For example, in regions with limited specialist access, frameworks could specify telemedicine-supported models or standardized escalation protocols for general practitioners and oncologists.

Importantly, such frameworks should avoid idealized care models that cannot be operationalized outside tertiary centers. Instead, they should prioritize actionable recommendations, such as minimum documentation standards, core competencies for initiating opioid therapy, and clearly defined referral thresholds, which can be reliably implemented across different settings [6].

Embedding these frameworks within digital clinical decision support tools could further promote adherence. Integration into electronic health records, with automated prompts for pain assessment and treatment escalation, has been shown to improve both documentation and prescribing in analogous contexts [13]. Combined with periodic audit and feedback cycles, these mechanisms would foster accountability and continuous improvement.

By harmonizing core standards while respecting regional constraints, these adaptable frameworks offer a realistic pathway to narrowing the implementation gap that currently limits the impact of both national legislation and international guidelines.

Collectively, these proposals represent a cohesive response to the intertwined barriers identified: educational deficits addressed through interdisciplinary training; fragmentation mitigated by integrated orientation centers; poor collaboration countered through structured exchanges; and uneven practice corrected via shared, adaptable frameworks. Implementing these initiatives will require sustained commitment from professional societies, academic institutions, and health authorities alike. Crucially, they place pain management at the center of oncology practice—not as an adjunct, but as an essential determinant of patient outcomes and quality of life [11,14].

While this analysis focused specifically on the Italian healthcare context, reflecting the scope and expertise of the participating societies, many of the identified barriers mirror challenges reported internationally. Educational gaps in pain management, fragmented care pathways, unclear professional roles, and implementation difficulties with standardized protocols have been documented across diverse healthcare systems [7,8,11,14]. However, the proposed solutions were developed within the framework of the Italian NHS and regional healthcare organization. Their adaptation to other countries would require consideration of differences in medical education curricula, healthcare delivery models, regulatory frameworks, and resource allocation mechanisms. Nevertheless, the core principles underlying our recommendations—interdisciplinary education, integrated care coordination, role clarification, and adaptable implementation strategies—may offer transferable insights for healthcare systems facing similar challenges in cancer pain management.

## 4. Toward Implementation and Advocacy

Achieving timely, integrated, and effective pain management for patients with cancer demands not only clinical refinement but also systemic and policy-level transformation. This document reaffirms a collective commitment to translating evidence and shared proposals into sustained, coordinated action. Central to this effort is the integration of structured, interdisciplinary pain education across all levels of medical training, thereby equipping clinicians with the competencies needed to deliver guideline-concordant care [9]. Equally essential is the reorganization of clinical pathways to support collaborative, patient-centered models, ensuring that expertise in oncology, pain medicine, and palliative care is deployed proactively rather than reactively [13].

Supporting these efforts, the inclusion of chronic cancer-related pain as a distinct diagnostic category (MG30.1) in the ICD-11 classification system represents a significant advancement in improving pain visibility in health statistics and promoting collaborative management [15,16]. This enhanced classification supports our proposed systematic approach by enabling better documentation and resource allocation decisions across healthcare settings.

Beyond the clinical domain, institutional and policy engagement will be indispensable. Effective implementation of standardized, adaptable therapeutic frameworks requires alignment with healthcare governance structures, robust resource allocation, and mechanisms to ensure accountability [3]. Advocacy efforts should also focus on addressing systemic barriers, such as uneven regional infrastructure and regulatory complexities, which perpetuate disparities in pain care [6].

## 5. Conclusions

Ultimately, improving cancer pain management necessitates a dual approach: advancing professional competencies through education and interdisciplinary practice, while simultaneously mobilizing policy instruments to enable sustainable integration of best practices into routine care. By reinforcing these complementary strategies, the pathway toward equitable, effective pain control in oncology becomes actionable. Such progress is not only essential for alleviating suffering but represents a fundamental imperative for comprehensive, value-driven cancer care.

## Figures and Tables

**Table 1:** Key barriers to effective cancer pain management and corresponding proposed solutions.

Identified Barrier/Gap	Proposed Solution
Insufficient education in pain management	Expand interdisciplinary training; integrate pain and palliative care modules into undergraduate and specialty curricula; and deliver joint educational webinars and case-based workshops [4,9,11].
Fragmented care and lack of interdisciplinarity	Establish integrated oncology orientation centers; implement routine multidisciplinary meetings; and enable co-assessment of more complex patients [3,13].
Limited mutual understanding of professional roles	Promote cross-disciplinary congress sessions, joint educational initiatives, collaborative workshops, and clinical case discussions to clarify roles and foster team-based care [4,13].
Challenges in implementing PDTAs	Develop adaptable national therapeutic frameworks/guidelines; embed pain assessment prompts into digital records; align protocols with local resources; and enable integration of telemedicine tools [3,6].
Patient-related barriers and misconceptions	Implement structured patient education programs; develop shared decision-making tools; address opioid misconceptions through clear communication strategies; and engage patients as partners in pain management [15].

PTDAs, diagnostic and therapeutic care pathways.

## Data Availability

No new data were created or analyzed in this study.
